# Crystallization Mechanism in Spark Plasma Sintered Bulk Metallic Glass Analyzed using Small Angle Neutron Scattering

**DOI:** 10.1038/s41598-020-58748-3

**Published:** 2020-02-06

**Authors:** Tanaji Paul, Ashish Singh, Kenneth C. Littrell, Jan Ilavsky, Sandip P. Harimkar

**Affiliations:** 1 School of Mechanical and Aerospace Engineering, Oklahoma State University, Stillwater, OK 74078 United States; 2 X-ray Science Division, Advanced Photon Source, Argonne National Laboratory, 9700 South Cass Avenue, Argonne, IL 60439 United States; 3 Present Address: Welspun Pipes Inc., 9301 Frazier Pike, Little Rock, AR 72206 United States; 4 Neutron Scattering Division, Oak Ridge National Laboratory, Oak Ridge, TN 37831 United States

**Keywords:** Mechanical engineering, Glasses, Metals and alloys, Characterization and analytical techniques

## Abstract

Understanding the thermal stability of metallic glasses is critical to determining their safe temperatures of service. In this paper, the crystallization mechanism in spark plasma sintered Fe_48_Cr_15_Mo_14_Y_2_C_15_B_6_ metallic glass is established by analyzing the crystal size distribution using x-ray diffraction, transmission electron microscopy and *in-situ* small angle neutron scattering. Isothermal annealing at 700 °C and 725 °C for 100 min resulted in the formation of (Fe,Cr)_23_C_6_ crystals, measured from transmission electron micrographs, to be from 10 to 30 nm. The small angle neutron scattering intensity measured *in-situ*, over a Q-range of 0.02 to 0.3 Å^−1^, during isothermal annealing of the sintered samples, confirmed the presence of (Fe,Cr)_23_C_6_ crystals. The measured scattering intensity, fitted by the maximum entropy model, over the Q-range of 0.02 to 0.06 Å^−1^, revealed that the crystals had radii ranging from 3 to 18 nm. The total volume fraction of crystals were estimated to be 0.13 and 0.22 upon isothermal annealing at 700 °C and 725 °C for 100 min respectively. The mechanism of crystallization in this spark plasma sintered iron based metallic glass was established to be from pre-existing nuclei as confirmed by Avrami exponents of 0.25 ± 0.01 and 0.39 ± 0.01 at the aforesaid temperatures.

## Introduction

Iron based metallic glasses constitute an advanced group of materials that have engrossed a major share of modern scientific research by virtue of their exciting properties and the multitude of potential applications^1^. These materials are solid alloys of about 80 atomic percent metal, principally Fe with Cr, Mn, Co, Ni, Zr, Nb and Mo, along with metalloids such as B, C, Si and P^2^. Fe-Si-B^3^, Fe-Cr-Zr-B^4^, Fe-Mo-C-P-B^5^, Fe-Co-Ni-Zr-B^6^ and Fe-Mn-Mo-Cr-C-B^7^ are some of the examples of iron based alloy systems reported to form metallic glasses. Structurally, they exhibit a dense disordered atomic structure^8,9^ that is the primary reason for their superior properties. For example, the yield strength of Fe_80_B_20_ metallic glass ribbons is as high as 3.6 GPa^10^. Hardness of iron based metallic glasses are also superior, about 12 GPa^11^, as compared to that of conventional polycrystalline steels. Metallic glasses also exhibit enhanced corrosion resistance at a much lower Cr content than stainless steels. For instance, Fe_72_Cr_8_P_13_C_7_ metallic glass ribbons hardly show any detectable corrosion rate in 1 N NaCl solution at ambient temperature^12^. Crystalline austenitic stainless steels with more than twice the Cr content, in contrast, corroded at a rate of about 1 mm/year. Melt spun metallic glass ribbons in the Fe-Si-B alloy system also possess high permeability and low core loss of about one-third that of grade M3 electrical steel. *Metglas*^R^ 2605SA1 ribbons exhibit saturation induction of 1.56 T and a maximum dc permeability of 600 kNA^−2^ ^13–15^. These properties, combined with the fact that iron based metallic glasses are significantly cheaper than other Zr or Pd based metallic glasses, have made these materials extremely versatile. As a result, they are employed in a wide variety of applications ranging from magnetic cores in distribution transformers to corrosion resistant thermally sprayed coatings^16–18^. By virtue of their high strength, iron based metallic glasses also harbor remarkable potential for structural applications.

In order to achieve the dense, disordered structure, metallic glasses are conventionally manufactured by rapid solidification processing^19^. This technique involves rapid quenching of the liquid melt of the metallic glass system into a solid alloy^20^. The primary criterion of processing a metallic glass can thus be understood to be one of rapid extraction of heat that eliminates the possibility of nucleation and growth of crystals within the molten alloy. The cooling rate necessary to achieve a glassy structure in Fe based alloy systems is almost 10^6^ Ks^−1^ ^21^. Since the rate of thermal conduction across two points is inversely proportional to the distance between them, it is imperative to reduce the thickness of the metallic glass to the order of few micrometres in the direction of heat extraction. This imposes a severe restriction on the forms into which metallic glasses can be manufactured, such as ribbons, wires and powder^22^.

For the cost effective application of iron based metallic glasses with high strength as structural components, it is necessary to circumvent the problem of processing these materials into bulk shapes. One of the promising methods is the application of powder consolidation techniques such as hot pressing^23^, warm extrusion^24^, hot isostatic pressing^25^ and spark plasma sintering (SPS)^26^. SPS is particularly advantageous as it enables the sintering of dense compacts at lower temperatures and shorter cycles^27–30^ primarily due to Joule heating resulting from the passage of current through the powder compact^31,32^. These advantages have led to the widespread applicability of SPS as means for prosessing difficult to sinter materials such as metallic glasses, ceramics and their composites^30^. For example, SPS was employed to process dense Fe_48_Cr_15_Mo_14_Y_2_C_15_B_6_ metallic glass compacts up to 20 mm at 550 °C within a short cycle time of 20 min^33^. Thus SPS holds significant promise for the processing of bulk sized iron based metallic glasses and hence attracts unabated attention in the research community.

Although powder compaction is a promising technique to process bulk metallic glasses, devitrification during service at elevated temperatures can result in deterioration of properties. Therefore, understanding the thermal stability of metallic glasses by investigating *in-situ* crystallization is critical to determining their safe temperatures of operation. Moreover, with the advent of a wide range of thermal processing techniques for the manufacturing of metallic glasses, their composites and coatings, the capability to predict the crystalline phases, their size and volume fraction is essential for determining optimized process parameters. Moreover, designing *in-situ* crystallized composites is a potential procedure to improve global plasticity of bulk metallic glasses^34^. For example, iron based metallic glass matrix, *in-situ* reinforced with *α*-Fe crystals exhibited an enhanced compressive plastic strain of 37.5% compared to 4% in monolithic one without compromising the elastic limit^35^. Hence the importance of the analysis of the mechanism and kinetics of thermally induced crystallization in metallic glasses for their successful processing and applications cannot be overemphasized.

Reported analyses on crystallization in spark plasma sintered iron based bulk metallic glasses suffer from drawbacks of the techniques employed. For example, calorimetric studies on *in-situ* iron based bulk metallic glass matrix composites^36^ only provided limited information on the activation energy of the exothermic reactions that is largely dependent of the analytical model used^37,38^. While crystal sizes can be estimated from x-ray diffraction, it is unexpected that the evolved crystals would exhibit a single size^39^. It has been proven that the mechanical properties and thermal stability of these metallic glasses are dependent not only on the mean size of the crystals but also on their distribution and volume fraction^40^. In this regard, small angle neutron scattering (SANS) is a powerful technique for an accurate quantitative analysis of crystallization in spark plasma sintered iron based bulk metallic glasses^41^. It enables the acquisition of a high intensity of scattering from crystals embedded within a metallic glass matrix *in-situ* during annealing. This intensity can be acquired over a broad range of the scattering vector, Q and hence facilitate the study of the evolution of structure across a wide span of length scales^42^, D from the direct relationship between them as D = 2*π*/Q^43^. The small angle scattering analysis tool IRENA^44^ complements this with the capability of calculating scattering contrast of any phase as a function of its composition and can be utilized to obtain an accurate estimate of the distribution of size and volume of each crystal. SANS equipped with supporting structural and microscopic techniques can thus be utilized to establish a cogent understanding of the crystallization in metallic glasses.

This paper thus aims to analyze the crystallization behavior in spark plasma sintered Fe_48_Cr_15_Mo_14_Y_2_C_15_B_6_ bulk metallic glass during annealing. Development of the microstructure during isothermal annealing is presented. The SANS intensity acquired during annealing is fitted with a size distribution tool to estimate the evolution of the diameter and volume of crystals. These results are utilized to establish the mechanism of crystallization in this material.

## Results and Discussion

### Structural analysis

**Figure 1:**
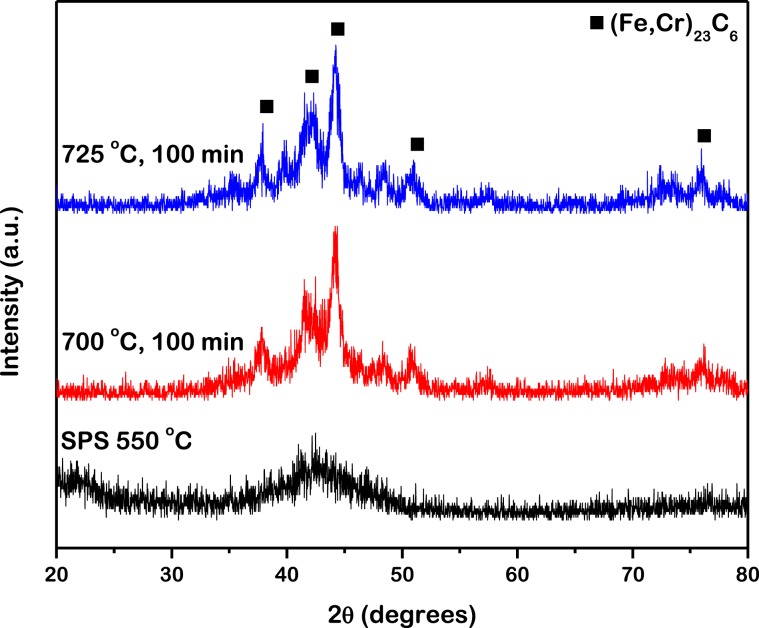
X-ray diffraction spectra of the iron based metallic glass spark plasma sintered at 550 °C and then annealed at 700 °C and 725 °C for 100 min each. The sintered sample retained a fully amorphous structure while the annealed ones developed ((Fe,Cr)_23_C_6_) crystals.

The x-ray diffraction (XRD) spectrum of the iron based metallic glass spark plasma sintered at 550 is presented in Fig. 1. It only exhibits a diffused peak, characteristic of fully amorphous materials whereas sharp peaks, indicative of crystalline phases, are absent. Thus it can be concluded that upon spark plasma sintering (SPS) at a temperature of 550 °C, the iron based metallic glass sample retained an amorphous structure. The differential scanning calorimetric thermogram of this iron based metallic glass manifests a glass transition temperature, Tg of about 575 °C and a crystallization onset temperature, Tx of about 653 °C^45^. Thus it is comprehensible that the sample sintered at 550 °C, a temperature below Tg, remains in the glassy state. The XRD spectra of the iron based metallic glass spark plasma sintered at 550 °C and then annealed at 700 °C and 725 °C for 100 min each, are also presented. In contrast to solely a broad peak, these spectra exhibit additional peaks, superimposed on the amorphous background, characteristic to crystals evolved within the iron based metallic glass matrix. The temperatures of annealing (700 °C and 725 °C) are considerably higher than the T_x_ of this metallic glass which resulted in the evolution of these crystals.

The peaks corresponding to the crystals, identified to be complex carbides of (Fe,Cr)_23_C_6_^41,46^, are observed to exhibit significant broadening which are suggestive of their small sizes. The size of crystals evolved from an amorphous matrix of iron based metallic glass upon thermal processing can be approximately estimated according to the Scherrer equation expressed as^47^:

1 $$D=\frac{0.9\lambda }{\beta cos{\theta }_{B}}$$

where D (Å) is the size of the crystals, *λ* (Å) is the wavelength of x-rays, *β* (rad) is the integrated breadth of the Bragg peak and $\theta$_B_ (°) is the Bragg angle. The estimated size of crystals according to Eq. 1 is about 9 nm for samples annealed at both 700 °C and 725 °C for 100 min. This result is consistent with previous observations where the size of (Fe,Cr)_23_C_6_ crystals evolved during annealing at 700 °C for 180 min estimated by the Scherrer equation was reported to be about 16 nm^46^. However, a number of limitations in employing the Scherrer equation must be noted. In utilizing Eq. 1, the shape factor, size factor, strain factor and preferred orientation factor have not been accounted for. As a result, Eq. 1 yields only an approximate estimate of the crystal dimensions as opposed to an accurate size. Moreover, the evolution of crystals in a metallic glass due to annealing is a complex phenomenon driven by nucleation and growth of a number of concentration gradients^41^. It is unlikely that such a phenomenon would result in a monodispersed size distribution of crystals in the metallic glass matrix, as estimated from Eq. 1. This distribution in size of crystals was investigated further by microstructural characterization as detailed in the following section. Fe_3_Mo_3_C has also been reported to have evolved during annealing of this metallic glass at 700 °C and above^48^. However, presence of this phase could not be detected from the XRD spectra in the present investigation.

### Microstructural characterization

**Figure 2:**
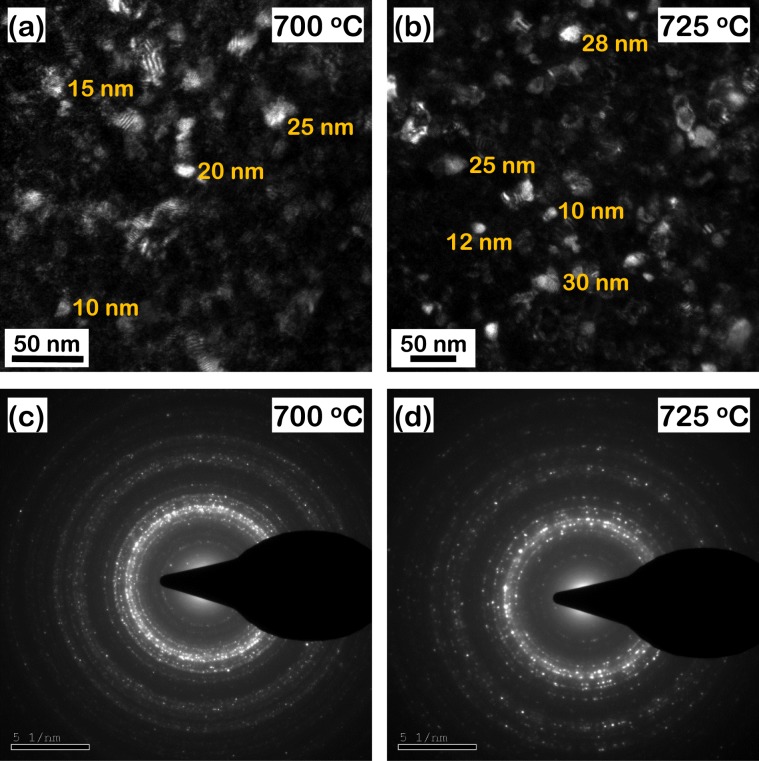
(**a,b**) Transmission electron microscope dark field images and (**c,d**) corresponding selected area diffraction patterns of spark plasma sintered iron based metallic glass annealed at 700 °C and 725 °C for 100 min. Regions of brighter contrast in (**a**,**b**) represent crystals embedded within the metallic glass matrix that exhibit a range of diameter from 10 to 30 nm.

The transmission electron microscope (TEM) dark field images of the SP sintered iron based metallic glass samples annealed at 700 °C and 725 °C for 100 min are presented in Fig. 2(a,b) respectively. The regions of brighter contrast depict the crystals evolved due to annealing from the metallic glass matrix depicted by the regions of darker contrast. The crystals are observed to be distributed homogeneously throughout the metallic glass matrix. Diameter of the individual crystals were measured from these dark field TEM images by ImageJ with some representative measurements presented herewith. As discussed in the previous section, in contrast to a monodispersed size distribution, the crystals exhibit a range of diameters from about 10 nm to about 30 nm. However, the relative volume fractions of the individual crystal sizes distributed throughout the entire samples cannot be obtained by this manual measurement technique.

The corresponding selected area diffraction patterns (SADPs) of the annealed samples are presented in Fig. 2(c,d). The diffuse rings observed in the patterns confirm that a significant volume fraction of the amorphous metallic glass matrix is retained even after annealing. The remaining volume fraction, evolved into crystals of (Fe,Cr)_23_C_6_ resulting in the diffraction spots superimposed on the diffuse rings. A detailed quantitative estimate of the size distribution of crystals and their relative volume fractions is obtained from the small angle neutron scattering (SANS) analysis presented in the following sections.

### Evolution of structure based on *in-situ* small angle neutron scattering

**Figure 3:**
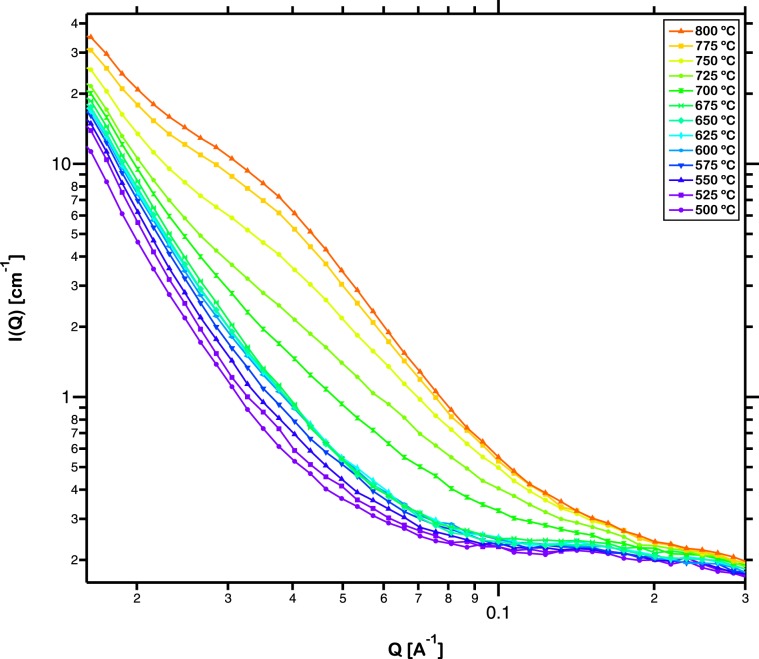
Log-log plot of *in-situ* small angle neutron scattering intensity measured over a Q-range of 0.02 to 0.3 Å^−1^ for the sintered sample continuously annealed from ambient temperature to 800 °C. Extremely low variation in intensity was observed below 500 °C and is not presented here.

The structural evolution of the iron based metallic glass was quantitatively analyzed by *in-situ* SANS during annealing. Figure 3 presents the SANS intensity over a Q-range of 0.02 to 0.3 Å^−1^ in a log-log plot for the sintered sample continuously annealed from ambient temperature to 800 °C. Extremely low variation in intensity was observed below 500 °C and is not presented here. The characteristics of scattering, corresponding interpretation as representative of phenomena occurring over distinct length scales and their evolution with increase in annealing temperature are discussed across three discrete Q-ranges as follows.

First, over the low Q-range below 0.02 Å^−1^, scattering indicates the presence of structural inhomogeneities larger than 30 nm that could possibly be Y and Mo-rich precipitate free zones (PFZs) embedded within the metallic glass matrix^49^. These PFZs have been observed during annealing of as-cast iron based metallic glass with diameter up to 200 nm^46^. They develop from the metallic glass matrix and upon further enrichment by Y possess an increased glass forming ability (GFA)^50^. Thus they retain their amorphous nature and are hence not detectable in the XRD spectrum (Fig. 1). The PFZs have high thermal stability and thus continue to contribute to the scattering intensity over this Q-range at higher temperatures up to 800 °C^46^. The neutron scattering length densities, *ρ* (m^−2^) of the PFZs and (Fe,Cr)_23_C_6_ and their corresponding contrasts, Δ*ρ*^2^ (m^−4^) with respect to the Fe_48_Cr_15_Mo_14_Y_2_C_15_B_6_ metallic glass matrix can be calculated based upon their reported composition^48^. The composition of the PFZs at low temperatures is Fe_41_Cr_16_Mo_29_Y_14_ (at.%). They get enriched in Y and Mo and depleted of Fe and Cr with increase in temperature and has a composition of Fe_35_Cr_11_Mo_37_Y_17_ at 700 °C. This fact is incorporated while calculating the scattering contrasts, presented in Table 1. The composition of (Fe,Cr)_23_C_6_ remains fairly constant with temperature. It can be seen that the scattering contrast of the Y and Mo-rich PFZs is always higher than that of (Fe,Cr)_23_C_6_. This results in a higher measured intensity at the Q-range characteristic of the PFZs (below 0.02 Å^−1^) than that at the Q-range characteristic of the (Fe,Cr)_23_C_6_ carbides (from 0.02 to 0.2 Å^−1^). Moreover, the scattering contrast of the PFZs increases with temperature which is manifested in the increase in scattering intensity with temperature over the Q-range below 0.02 Å^−1^.

**Table 1:** Composition, Q range, neutron scattering length density, *ρ* and neutron scattering contrast, Δ*ρ*^2^ of the metallic glass matrix, PFZs and crystals.

Composition	Q range (Å^−1^)	*ρ* (x10^−14^ m^−2^)	Δ*ρ*2 (x10^−28^ m^−4^)
Metallic glass (Fe_48_Cr_15_Mo_14_Y_2_C_15_B_6_)	<0.02 Å^−1^	6.75	0
PFZ (Fe_41_Cr_16_Mo_29_Y_14_)	<0.02 Å^−1^	4.95	3.24
Y and Mo enriched PFZ (Fe_35_Cr_11_Mo_37_Y_17_)	<0.02 Å^−1^	4.69	4.25
Crystal ((Fe,Cr)_23_C_6_)	0.02 to 0.2 Å^−1^	7.28	1.15

Second, over the mid Q-range from 0.02 to 0.2 Å^−1^, scattering results from the evolution of (Fe,Cr)_23_C_6_ crystals within the iron based metallic glass matrix during continuous annealing. The diameter of the carbides is in the range of about 3 to 30 nm, in good agreement with those reported earlier^46^. The formation of the PFZs results in the depletion of Y content in the surrounding matrix that results in a reduction of the GFA. These regions are thus enriched in Fe and Cr eventually evolving into crystalline carbides. Figure 3 shows that significant increase in scattering intensity does not occur until 675 °C which exhibits the stability of this iron based metallic glass and its resistance to crystallization. Thereafter, with further increase in temperature beyond 700 °C, profuse crystallization occurs resulting in a monotonic increase in the scattering intensity in this Q-range.

Finally, over the high Q-range above 0.2 Å^−1^, background scattering occurred due to the presence of roughness at the sample surface, the detector and other minute variations. These contributions are unrelated to the evolution of crystals and are roughly independent of Q^51^. This is further supported by the fact that the background scattering does not vary with increment in the temperature of annealing.

### Crystal size distribution

**Figure 4:**
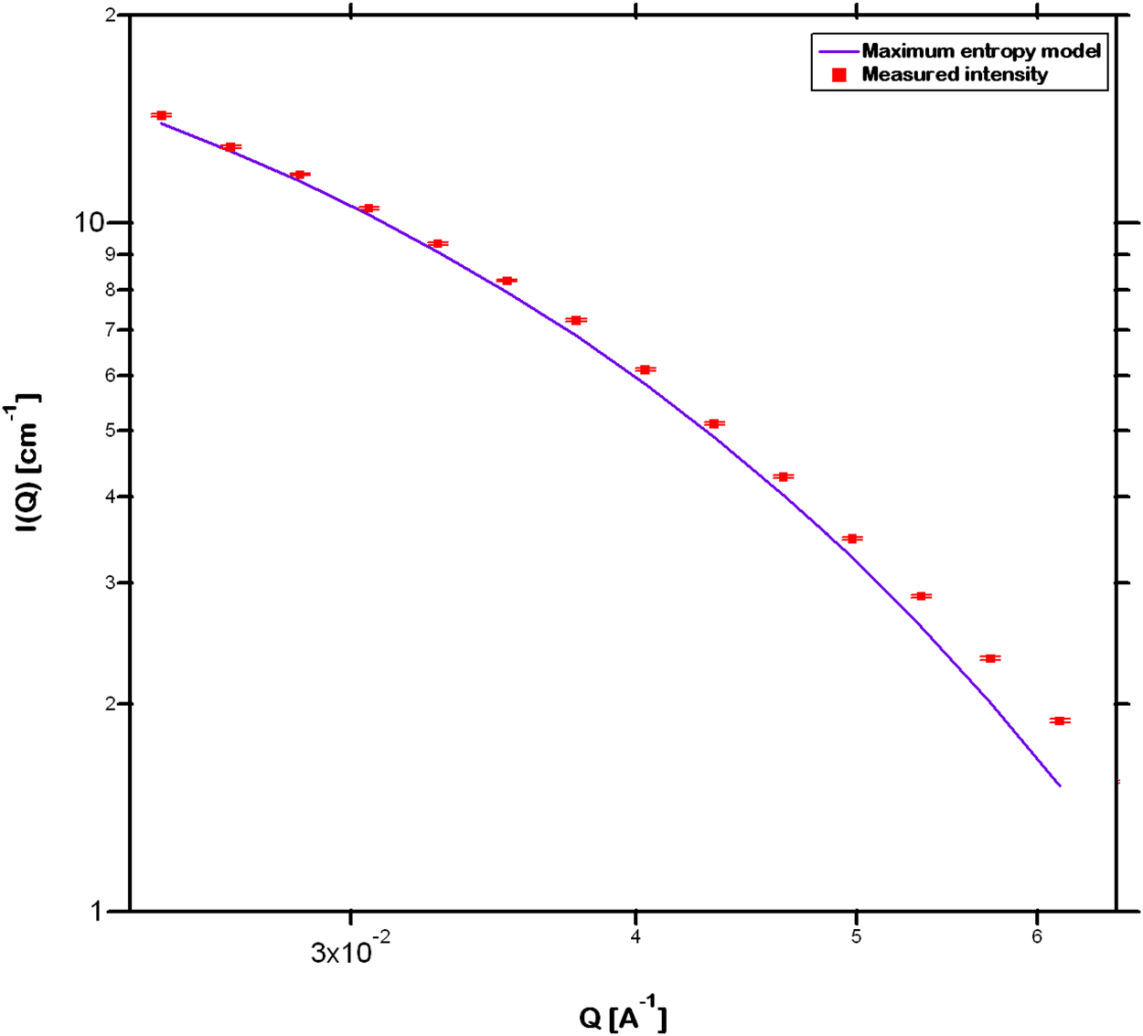
Representative fit of the maximum entropy model to the measured small angle neutron scattering intensity over a Q-range of 0.02 to 0.06 Å^−1^ utilized to analyze the distribution of size and volume fraction of (Fe,Cr)_23_C_6_ crystals. The fitted model agrees well with the measured data.

The SANS intensity was utilized to analyze the distribution of size and volume fraction of (Fe,Cr)_23_C_6_ crystals. The maximum entropy model^52^ in the size distribution tool available in IRENA software suite^44^ was employed for this purpose. Each scatterer of any size scatters at all Q-ranges and it is impossible to distinguish it from the background scattering from the pristine metallic glass, particularly in this multicomponent system. Hence, in order to eliminate possible error, only the mid Q-range, characteristic of the crystals whose size distribution is of primary interest here, is fitted. It is assumed in this model that the scattering particles are approximately equiaxed which is valid in the present case as can be observed from the TEM images presented in Fig. 2(a,b) where the growth of crystals is reasonably isotropic and no particular growth direction appears to be favored over another. Under this assumption the tool fits and thereby estimates the size distribution of spheres, each with a uniform diameter. Figure 4 presents a representative maximum entropy model fit to the measured intensity for the sample annealed at 800 °C. The measured intensity is higher than the fitted model at higher Q which suggests that there exists additional scattering, possibly from Fe_3_Mo_3_C, however of extremely small magnitude due to the much lower scattering contrast, 0.04 × 10^−28^ m^−4^ as compared to that of (Fe,Cr)_23_C_6_ (Table 1) with respect to the metallic glass. The size distribution analysis of various carbides including (Fe,Cr)_23_C_6_ and Fe_3_Mo_3_C evolved in steels as a result of annealing has been successfully performed and the results validated with microscopic characterization techniques^53^. This indicates that the magnetic contribution from these carbides does not affect the distribution of size and volume fraction estimated from fitting the SANS intensity. Additionally, since the experiments were carried out in the absence of a magnetic field the ferromagnetic coupling between the crystals is unlikely to affect the estimated size distribution^41^.

### Isothermal crystallization kinetics

**Figure 5:**
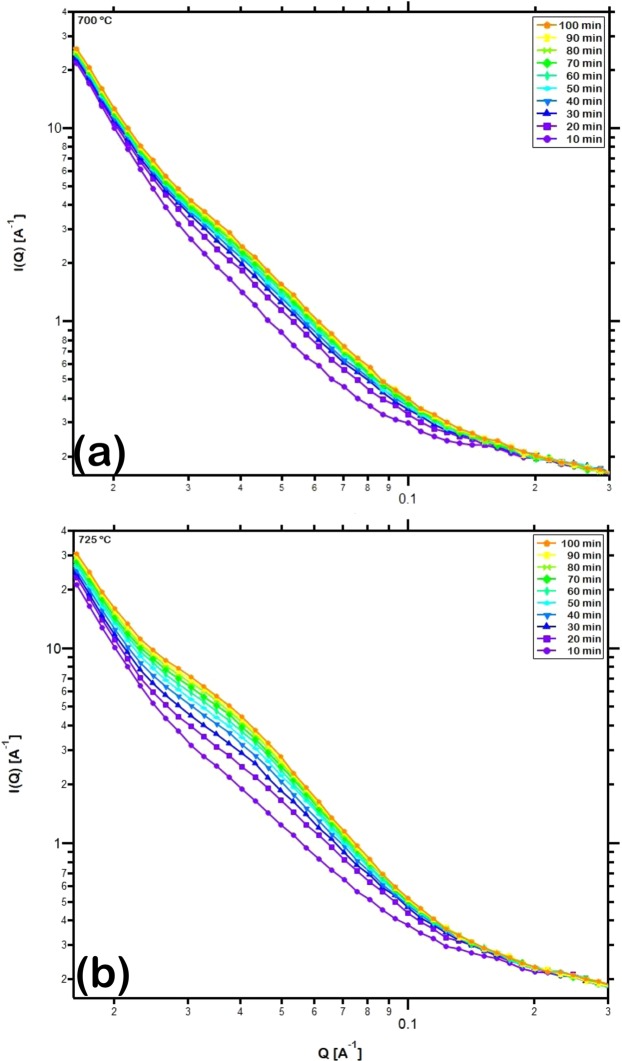
Log-log plot of *in-situ* small angle neutron scattering intensity measured over a Q-range of 0.02 to 0.3 Å^−1^ for the sintered sample isothermally annealed at (**a**) 700 °C and (**b**) 725 °C for 100 min.

**Figure 6:**
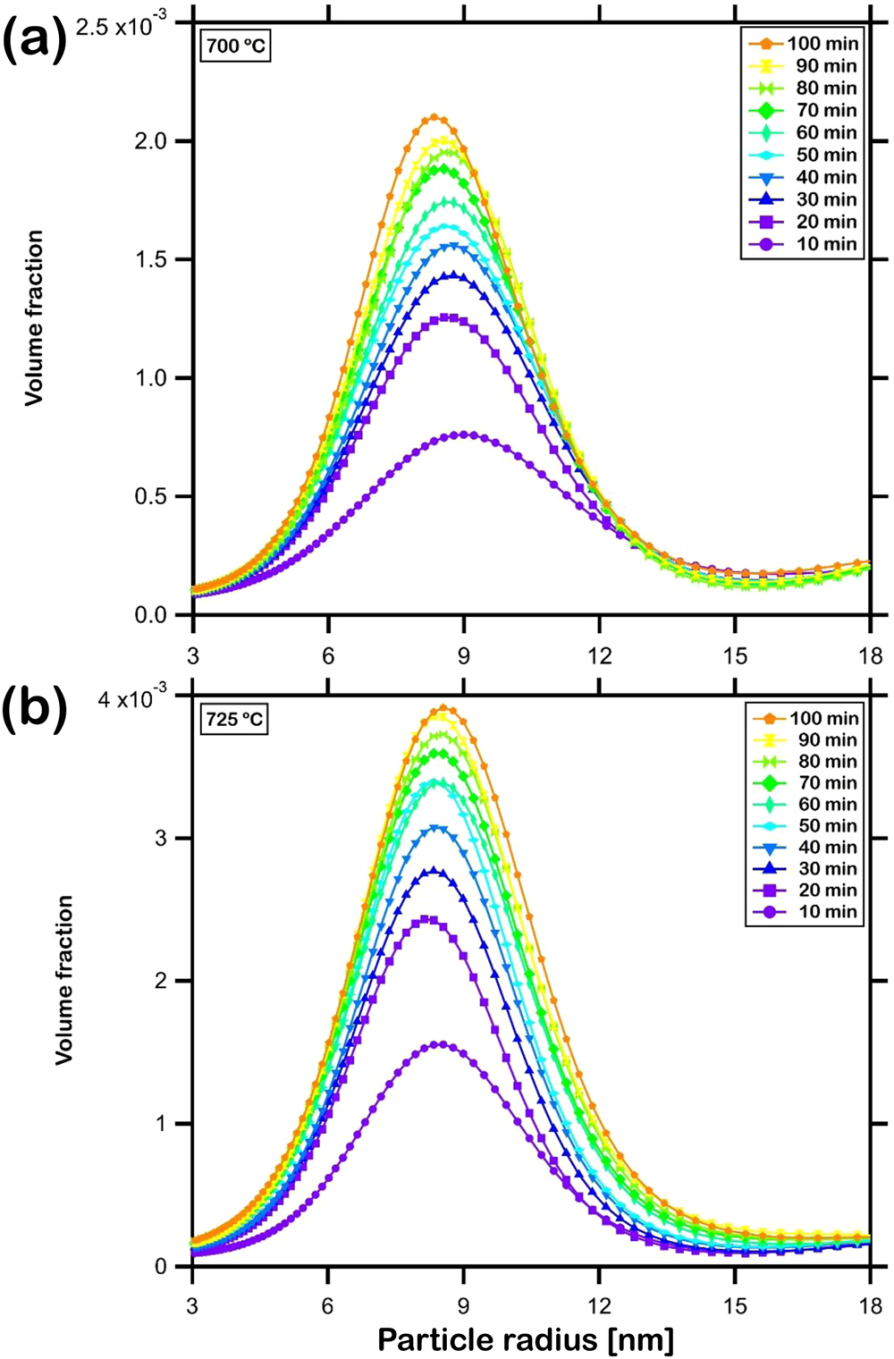
Distribution of the volume fraction of (Fe,Cr)_23_C_6_ crystals of various sizes estimated by fitting *in-situ* small angle neutron scattering intensity measured during isothermal annealing of the sintered sample at (**a**) 700 °C and (**b**) 725 °C for 100 min. The crystals exhibit a range of radii from 3 to 18 nm.

The isothermal crystallization kinetics of the samples were investigated at temperatures that exhibited observable variation in SANS intensity presented in Fig. 3 while being close to the crystallization onset temperature of this metallic glass. Accordingly, the evolution of the SANS intensity during isothermal annealing of the samples at 700 °C and 725 °C for 100 min is presented in Fig. 5. The higher intensity of scattering confirms the higher volume fraction of crystals evolved at the higher temperature of annealing. This measured intensity was fitted by the routine explained in the previous section to estimate the volume distribution of crystal sizes in the annealed metallic glass. The resulting distribution of (Fe,Cr)_23_C_6_ crystals of various radii is presented in Fig. 6. It can be observed that the crystals evolved have radii ranging from 3 to 18 nm, in agreement with the sizes observed in the TEM dark field images (Fig. 2(a,b)). Among them, crystals with radii around 8 to 9 nm are observed to be in highest abundance. With increase in duration of isothermal annealing, the volume fraction of all crystal sizes increases. The total volume fraction, *x* of (Fe,Cr)_23_C_6_ crystals during isothermal annealing increased from 0.07 to 0.13 at 700 °C and from 0.10 to 0.22 at 725 °C. It can also be observed from Fig. 6 that the increase in volume fraction of carbides evolved with time is rapid during the early stages of isothermal annealing and slows down during the later stages. These crystals develop preferentially in the regions depleted of Y and Mo and enriched in Fe and Cr and the rate of growth is rapid due to the large driving force resulting from the reduction in free energy from amorphous to crystalline state. With time, concentration gradients in regions neighboring these crystals grow and eventually encounter with one another, a process termed ‘soft impingement’, and suppress the rate of coarsening of the crystals^54^.

### Crystallization mechanism

**Figure 7:**
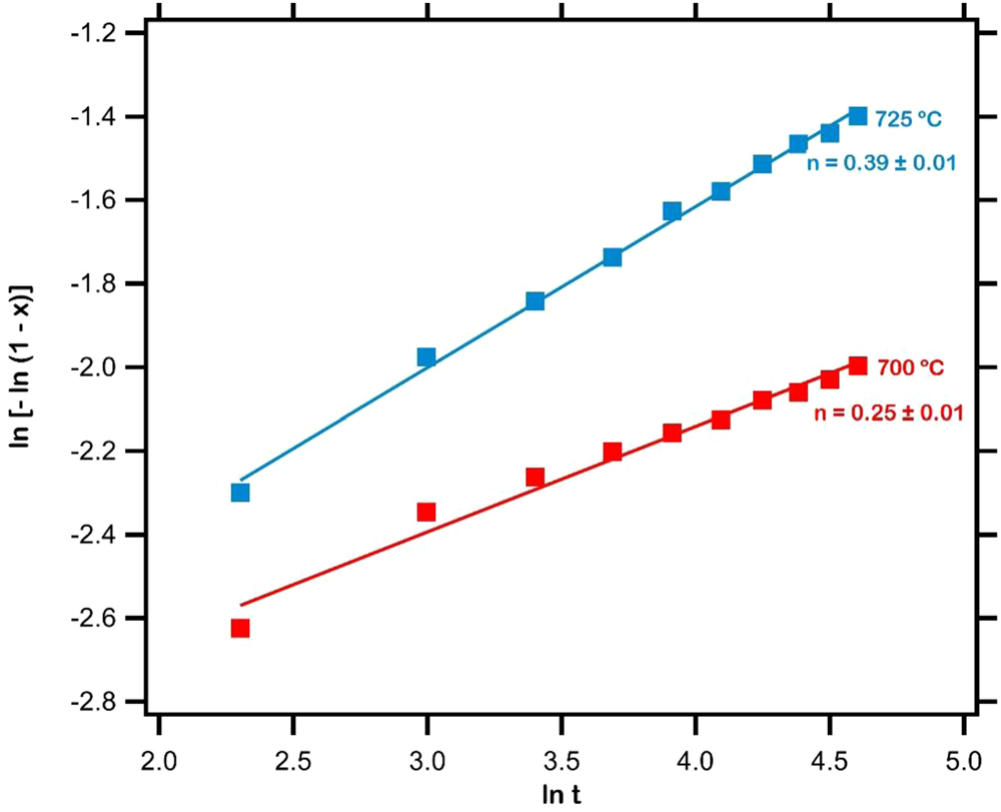
Plots of $\mathrm{ln}$[−$\mathrm{ln}$(1 − $x$)] versus $\mathrm{ln}\,t$ for the evolution of (Fe,Cr)_23_C_6_ crystals during isothermal annealing of Fe_48_Cr_15_Mo_14_Y_2_C_15_B_6_ metallic glass. The estimated Avrami exponents, 0.25 $\pm$ 0.01 and 0.39 $\pm$ 0.01 at 700 °C and 725 °C respectively, established that crystals evolved from pre-existing nuclei.

In order to establish the mechanism of isothermal crystallization in the spark plasma sintered iron based metallic glass, the results obtained from the *in-situ* small angle neutron scattering analysis of the distribution of volume fraction of (Fe,Cr)_23_C_6_ crystals were modeled by the Johnson-Mehl-Avrami equation, expressed as^55,56^:

2 $$\mathrm{ln}\,[\,-\,\mathrm{ln}(1-x)]=n\,\mathrm{ln}\,t+n\,\mathrm{ln}\,k$$

where *x* is the total volume fraction of crystals, n is the Avrami exponent, t (min) is the isothermal annealing time and k is the reaction rate constant. The plots of ln[−ln(1 − *x*)] with respect to ln*t* are presented in Fig. 7. The Avrami exponent n was estimated, by linear fit to the plots, to be 0.25 ± 0.01 and 0.39 ± 0.01 at 700 °C and 725 °C respectively.

The Avrami exponent is representative of the nucleation behavior prevalent during the progress of crystallization in metallic glass^57^. In particular, a value of n less than 1.5, as in the present case, shows that the evolution of (Fe,Cr)_23_C_6_ crystals within the iron based metallic glass during isothermal annealing both at 700 °C as well as 725 °C occurs by diffusional transformation from pre-existing nuclei^58,59^. This transformation occurs with the growth of these crystallites alongside a decreasing nucleation rate, thereby being a primarily growth controlled process^21^. It was observed from Fig. 3 that during annealing from ambient up to 700 °C and 725 °C the nuclei developed in the metallic glass matrix resulted in the increase in scattering intensity. Thus at the beginning of isothermal annealing at these temperatures nuclei of (Fe,Cr)_23_C_6_ are pre-existent. With the progress of annealing time, these pre-existing nuclei grow into well developed crystals, as manifested by the estimated values of the Avrami exponent.

## Conclusions

Fully amorphous spark plasma sintered Fe_48_Cr_15_Mo_14_Y_2_C_15_B_6_ metallic glass upon isothermal annealing at 700 °C and 725 °C for 100 min resulted in the evolution of (Fe,Cr)_23_C_6_ crystals. The size of these crystals was estimated to be 9 nm according to the Scherrer equation. Dark field transmission electron micrographs, however, revealed that the diameter of these crystals embedded within the metallic glass matrix ranges from 10 to 30 nm. The *in-situ* small angle neutron scattering intensity, measured from the sintered samples during isothermal annealing, was fitted by the maximum entropy model under the assumption of spherical crystals. The estimated distribution of crystal radii exhibited a range from 3 to 18 nm with those around 8 to 9 nm of the largest abundance. The total volume fraction of crystals increased with isothermal annealing time from 0.07 to 0.13 at 700 and from 0.10 to 0.22 at 725 °C. The mechanism of crystallization in this spark plasma sintered iron based metallic glass, analyzed under the theoretical framework of the Johnson-Mehl-Avrami model, exhibited an exponent of 0.25 ± 0.01 and 0.39 ± 0.01 during isothermal annealing at 700 °C and 725 °C respectively, a manifestation of the evolution of crystals from pre-existing nuclei in the metallic glass matrix.

## Methods

### Spark plasma sintering

The metallic glass powder of composition Fe_48_Cr_15_Mo_14_Y_2_C_15_B_6_ (at.%) consisted of mostly spherical particles, with a mean size of about 40 μm^26,33^. A graphite die was lined with a graphite foil along its internal wall of diameter 20 mm to facilitate movement of graphite punches within. Approximately 3 g of the powder was introduced into the die followed by pre-pressing with another punch under a pressure of 5 MPa. The die was wrapped around the outer wall with graphite felt secured with a graphite yarn. The entire assembly was placed within the furnace chamber of a spark plasma sintering unit (Thermal Technology LLC, SPS 10-3) and a thermocouple placed at the inner wall of the die was utilized for temperature measurement. The powder was sintered at a temperature of 550 °C attained at a heating rate of 100 °C min^−1^ and soaking time of 10 min under a constant uniaxial compressive pressure of 70 MPa. These experiments yielded samples of diameter 20 mm and thickness about 1 mm.

### XRD and TEM

Structural analysis of the sintered samples was performed using an X-ray diffractometer (PANalytical, PW 1830) operated with Cu-K$\alpha$ radiation ($\lambda$ = 1.5418 Å). Thin samples were prepared by focused ion beam technique (FEI, Nova 200 NanoLab) followed by microstructural analysis in a transmission electron microscope (FEI, Tecnai F20) operated at 200 kV. Size of individual crystals was measured from the micrographs using a public domain image processing software, ImageJ (available from the National Institute of Health, USA).

### *In-situ* small angle neutron scattering

*In-situ* neutron scattering experiments were performed during isochronal and isothermal annealing of the sintered samples measuring 20 mm in diameter and 1 mm in thickness placed in an enclosed chamber previously calibrated for temperature and connected to a constant supply of argon. For isochronal annealing, the sintered samples were heated from ambient temperature up to 800 °C while for isothermal ones, they were heated up to 700 °C and 725 °C and held at the respective temperatures for 100 min each. The heating rate employed for all the experiments was 10 °C min^−1^. Neutron scattering intensities were measured as a function of the scattering vector in the range of 0.01 to 0.4 Å^−1^, *in-situ* during annealing, by the General-Purpose Small-Angle Neutron Scattering Diffractometer at beamline CG-2 of the High Flux Isotope Reactor, Oak Ridge National Laboratory^60,61^. The measured intensities were corrected for background from the sample holder, sample thickness and transmission. These data were modelled and analyzed by the size distribution tool available in IRENA software^44^. Neutron scattering length densities and the corresponding neutron scattering contrasts of the materials were calculated based on their compositions by the scattering contrast calculator support tool, also available in IRENA.

## Data Availability

The data that support the findings of this study are available from the corresponding author upon reasonable request.
